# Genotype Characteristics and Hearing Phenotype Analysis of Newborns with Biallelic GJB2 Mutations: A 652-Case–Cohort Study

**DOI:** 10.3390/ijns11040110

**Published:** 2025-12-03

**Authors:** Jianjun Li, Bo Wu, Wenlan Liu

**Affiliations:** 1 The Newborn Diseases Screening Center, Shenzhen Maternity and Child Healthcare Hospital, Southern Medical University, Shenzhen 518000, China; lijianjun@szmch.net.cn; 2 Shenzhen Key Laboratory of Maternal and Child Health and Diseases, Shenzhen 518000, China

**Keywords:** GJB2 gene, newborns, biallelic mutation, genotype-phenotype correlation, hearing screening

## Abstract

This study aims to investigate the genotype characteristics of newborns with biallelic GJB2 mutations and their correlation with hearing phenotypes, providing a basis for clinical genetic counseling and hearing management. A retrospective study was conducted on 652 newborns with biallelic GJB2 mutations detected at the Newborn Diseases Screening Center of Shenzhen Maternal and Child Health Care Hospital from January 2022 to December 2024. The differences in mutation types, hearing screening, and diagnostic results were analyzed and compared between the homozygous and compound heterozygous mutation groups to assess their correlation with hearing phenotypes. Genotype analysis identified 543 cases of homozygous mutations, mainly the c.109G>A/c.109G>A genotype (98.90%). Compound heterozygous mutations were identified in 109 cases, with the majority being c.109G>A/c.235delC (76.15%). Following two-stage hearing screening, 227 (34.82%) of the 652 cases were referred, with bilateral failure accounting for the majority (81.94%) of these cases. The referral rates showed no significant difference between the homozygous (35.54%) and compound heterozygous (31.19%) groups (*p* > 0.05). The overall hearing loss detection rate was 6.90% (45/652); among these, eight infants who had initially passed the newborn hearing screening were later found to have hearing loss between 2.5 and 6 months of age. Among the 45 confirmed deaf children, hearing loss was mainly mild to moderate (87.50%), and profound deafness was only seen in the homozygous mutation group (10.29%, 7/68 ears). Most newborns with biallelic GJB2 mutations passed the two-stage hearing screening, and associated hearing loss was typically mild to moderate. Long-term auditory monitoring remains essential for all genetically confirmed infants to monitor late-onset progression.

What is Already Known on This Topic: Biallelic mutations in the GJB2 gene are one of the important causes of hereditary deafness. The c.109G>A and c.235delC mutations are common in East Asian populations. However, the genotype-phenotype correlation and the risk of late-onset hearing loss still need further investigation.

What This Study Adds: Most (65.18%) newborns with biallelic GJB2 mutations passed the two-stage hearing screening, and associated hearing loss was typically mild to moderate. However, long-term auditory monitoring remains essential for all genetically confirmed infants as they are all at risk for late-onset progression.

How This Study Might Affect Research, Practice, or Policy: Our findings underscore the clinical value of genetic testing in informing counseling and management for newborns with biallelic GJB2 mutations. Furthermore, they pave the way for refining universal newborn hearing screening by integrating genetic data to better identify infants at risk for late-onset hearing loss.

## 1. Introduction

Hearing impairment is one of the most common birth defects in newborns, profoundly affecting children’s language development, cognitive abilities, and social skills [1,2]. The GJB2 gene encodes connexin 26, a protein that plays a crucial role in signal transmission within the inner hair cells of the cochlea. Mutations in this gene are the most common cause of non-syndromic hereditary deafness [3]. To date, hundreds of GJB2 gene mutations have been reported worldwide, with significant clinical heterogeneity [4]. Some mutations (such as c.235delC and c.35delG) typically lead to severe to profound deafness [5], while others (such as c.109G>A) may present with milder hearing phenotypes or even normal hearing [6].

The widespread implementation of newborn hearing screening (NHS) has made it possible to detect hearing loss at an early stage. However, newborns carrying biallelic GJB2 mutations exhibit a wide range of hearing outcomes. Some infants may pass the initial screening but still develop late-onset or progressive hearing loss later on [7]. In recent years, the increasing application of genetic testing technologies has broadened the spectrum of the association between GJB2 mutations and hearing loss. However, the impact of different mutation types on hearing screening and diagnostic outcomes remains unclear, especially in large-scale studies of the newborn population.

Based on a cohort of 652 newborns carrying biallelic GJB2 mutations, this study systematically analyzed their mutation spectrum, hearing screening, and diagnostic results, and explored the correlation between different genotypes and hearing phenotypes. Our findings demonstrated that the c.109G>A homozygous genotype predominated among newborns with biallelic GJB2 mutations. Among these infants, only a small proportion (6.9%, 45/652) were diagnosed with hearing loss. The majority of affected infants (87.5%) exhibited mild to moderate hearing loss, and profound loss occurred exclusively in infants harboring homozygous mutations.

## 2. Materials and Methods

### 2.1. Study Subjects

This study enrolled 652 newborns with biallelic GJB2 mutations who were identified at the Newborn Diseases Screening Center of Shenzhen Maternal and Child Health Care Hospital between January 2022 and December 2024. All participants received audiological evaluations according to a standardized clinical protocol, which included initial screening, rescreening for those who did not pass, and diagnostic testing for those who continued to be referred. The study was approved by the Ethics Committee of Shenzhen Maternal and Child Health Care Hospital (Approval No.: SFYLS [2024]073), and informed consent was obtained from the guardians of all participants.

### 2.2. Dried Blood Spot Preparation and DNA Extraction

The national newborn screening protocol requires participating obstetric institutions to collect capillary blood samples from newborns within 48 h after birth using a standardized heel prick method. Three standardized dried blood spot (DBS) samples (diameter ≥ 8 mm) were prepared on dedicated filter paper according to the recommended collection procedures. These samples were maintained at 2–8 °C in a validated refrigeration system and transported to the Newborn Diseases Screening Center of Shenzhen Maternal and Child Health Care Hospital via a certified cold chain logistics system.

Genomic DNA was extracted from dried blood spot samples using the Ex-DNA Dried Blood Spot Genomic DNA Extraction Kit (Xi’an Tianlong Technology Co., Ltd., Xi’an, China) and the NP968-C Nucleic Acid Automatic Extraction System (Xi’an Tianlong Technology Co., Ltd., Xi’an, China), following the manufacturer’s instructions. The specific steps are as follows: 3–5 pieces of dried blood spot samples (6 mm × 6 mm) were placed in 300 µL of digestion buffer containing proteinase K and incubated at 56 °C for 20 min. Subsequently, 60 µL of nucleic acid release agent was added to facilitate DNA release. The automated extraction procedure included lysis at 80 °C for 10 min, three washing steps at 0–90 °C, and elution at 90 °C for 7 min. After extraction, the elutant was transferred to a nuclease-free centrifuge tube and stored at −20 °C for subsequent use.

### 2.3. Genetic Screening for Deafness-Related Gene Hotspot Mutations

Genomic DNA was extracted from dried blood spot samples using the Ex-DNA Dried Blood Spot Genomic DNA Extraction Kit (Xi’an Tianlong Technology Co., Ltd.), and then the 23 mutation hotspots in four common deafness-related genes (GJB2, SLC26A4, MT-RNR1, and GJB3 [c.538C>T]) were detected using a certified Neonatal Deafness Gene Screening Kit and the LuxScan 10K/D Gene Chip Scanner (Chengdu Boao Jingxin Biotechnology Co., Ltd., Chengdu, China). The screened mutation hotspots were as follows: GJB2 (c.35delG, c.109G>A, c.235delC, c.299_300delAT, c.176_191del16, c.257C>G, c.35insG, c.427C>T, c.512insAACG); SLC26A4 (c.919-2A>G, c.1174A>T, c.1226G>A, c.1229C>T, c.1707 + 5G>A, c.1975G>C, c.2027T>A, c.2168A>G, c.281C>T, c.589G>A, c.917insG); MT-RNR1 (m.1494C>T, m.1555A>G); and GJB3 (c.538C>T). All analytical procedures were strictly conducted in accordance with the manufacturer’s instructions.

### 2.4. Audiological Evaluation

The neonatal hearing screening protocol employed a two-stage, evidence-based approach. Initial screening was conducted using transient evoked otoacoustic emissions (OAE) with the OtoRead OAE detector (Middelfart, Denmark) within 48 h after birth, prior to hospital discharge. Infants who did not pass the initial screening were rescreened at 42 days of age using a combination of transient evoked OAE and automated auditory brainstem response (AABR) testing, performed with the 1077 AccuScreen ABR audiometer (Middelfart, Denmark). Diagnostic hearing assessment was performed using the Navigator PRO Auditory Brainstem Response system (Pleasant Prairie, WI, USA). For infants who failed the rescreening, this was completed within 3 months of age, while for those who passed, it was conducted upon parental request following concerns about hearing. Hearing diagnosis was based on the 2021 World Health Organization (WHO) grading criteria for hearing loss, which classifies hearing impairment into four grades according to the auditory brainstem response threshold as follows: mild (20–35 dB HL), moderate (35–65 dB HL), severe (65–80 dB HL), and profound (80–95 dB HL).

### 2.5. Follow-Up Protocol

Upon issuance of the genetic test report, guardians of infants with biallelic GJB2 mutations were contacted by telephone to determine their hearing screening results. Infants who did not pass the initial screening were referred to the Otolaryngology Department of our hospital for a comprehensive audiological diagnostic assessment at 3 months of age. Those who passed the screening were managed as part of a high-risk group for hearing loss. Telephone follow-ups were conducted to inform their guardians of the risk of delayed-onset or progressive hearing loss, provide guidance on observing auditory behavior at home, and recommend prompt return for a comprehensive audiological evaluation if any abnormalities were noted. Behavioral observation audiometry served as the core method for monitoring the hearing status of these infants.

### 2.6. Statistical Analysis

Statistical analysis was performed using IBM SPSS 26.0 software (IBM Corp., Armonk, NY, USA). Categorical variables were presented as frequencies and percentages (n [%]). Differences in hearing screening pass rates among mutation groups were compared using the chi-square test. A two-sided *p*-value of less than 0.05 was considered statistically significant.

## 3. Results

### 3.1. Genotype Distribution

A total of 652 newborns with biallelic GJB2 mutations were enrolled in this study. Among them, 543 cases had homozygous mutations, with the predominant type being c.109G>A/c.109G>A, accounting for as high as 98.90% (537/543). Compound heterozygous mutations were identified in 109 cases. The most prevalent genotype was c.109G>A/c.235delC, accounting for 76.15% (83/109) of these cases. The distribution of genotypes and mutations was shown in Table 1.

### 3.2. Hearing Screening Results

All 652 cases received hearing screening. The overall pass rate was 65.18% (425/652), meaning 227 cases failed the screening and were consequently referred for further diagnosis. Among these referred cases, 193 were from the GJB2 homozygous mutation group (35.54% of 543), and 34 were from the compound heterozygous mutation group (31.19% of 109). Chi-square analysis showed no statistically significant difference in the referral rates between these two groups (χ^2^ = 0.85, *p* = 0.357). Of note, bilateral referral was predominant in both groups, while no significant differences were observed for referral rates between the groups in either unilateral (*p* = 0.613) or bilateral (*p* = 0.218). Detailed hearing screening results are presented in Table 2.

### 3.3. Hearing Diagnosis Results

#### 3.3.1. Incidence of Hearing Loss

The overall detection rate of hearing loss was 6.90% (45/652). In the homozygous mutation group, the rate was 7.00% (38/543), and in the compound heterozygous mutation group, it was 6.42% (7/109). There was no statistically significant difference between these two groups (*p* = 0.884). Hearing loss was predominantly bilateral. Detailed hearing diagnosis results are shown in Table 3.

It was particularly noteworthy that among the 425 newborns who passed the hearing screening, subsequent follow-up identified 8 cases (1.88%) who were diagnosed with hearing loss between 2.5 and 6 months of age. In the homozygous mutation group, 6 cases were diagnosed with hearing loss at 2.5, 2.5, 2.5, 3, 3, and 6 months of age, respectively. In the compound heterozygous mutation group, 2 cases were diagnosed at 2.5 and 4 months of age, respectively. Detailed results of hearing screening and diagnosis were shown in Table 4.

#### 3.3.2. Severity of Hearing Loss

Among the 80 ears with confirmed hearing loss, the majority exhibited mild to moderate hearing loss, accounting for 87.50%. In the homozygous mutation group, this proportion was 86.76%, while in the compound heterozygous mutation group, it was 91.67%. All seven ears with profound hearing loss were found in the homozygous group. Specifically, the c.109G>A and c.235delC homozygous mutations accounted for four ears (in two individuals) and three ears (in two individuals), respectively. No cases of profound loss were detected in the compound heterozygous group. The distribution of hearing loss severity among GJB2 Genotypes was shown in Table 5 and Table 6.

## 4. Discussion

This study conducted a genotype-phenotype correlation analysis of 652 newborns with GJB2 biallelic mutations, yielding the following key findings: (1) the homozygous mutations were predominantly c.109G>A/c.109G>A (98.90%), while the most common compound heterozygous mutation was c.109G>A/c.235delC (76.15%); (2) the overall hearing screening failure rate was 34.82%, with no significant difference between the homozygous and compound heterozygous mutation groups; (3) the total detection rate of hearing loss was 6.90%, with 8 cases (17.78%) passing initial hearing screening but being subsequently diagnosed; (4) among confirmed cases, hearing loss was primarily mild-to-moderate (87.50%), with profound deafness observed exclusively in the homozygous mutation group. Our findings define the audiological profile of children with biallelic GJB2 mutations and underscore the necessity of integrating genetic screening with sustained audiological follow-up for the early detection of hearing loss.

Our findings that in the homozygous mutation group, the GJB2 c.109G>A homozygous mutation is overwhelmingly predominant (98.90%) are remarkably consistent with the findings of Ruan Y et al. (2024) and Zou Y et al. (2019) in Chinese multicenter cohorts (98.11% and 99.34%, respectively) [8,9]. The notably high frequency of the GJB2 c.109G>A variant in Asian populations suggests that it likely arose from a common ancestral origin and was subsequently propagated by a founder effect, possibly coupled with local positive selection pressures. However, the precise evolutionary mechanisms, including the potential selective advantages it might have conferred and the initial timing and dissemination of this mutation, remain to be fully elucidated and warrant further investigation. The most recurrent combination was the c.109G>A/c.235delC compound heterozygous variant, accounting for 76.15% of such cases. This finding is in line with the documented predominance of the c.109G>A and c.235delC alleles in the Chinese population [10,11]. The high prevalence of the c.109G>A/c.235delC genotype in our study was anticipated, given that the c.235delC mutation is the second most prevalent pathogenic variant in the Chinese population, with a reported allele frequency of 0.897% in a large-scale Beijing newborn screening study (n = 21,006) [12].

The overall hearing screening failure rate in this study was 34.82%, which was lower than the 56.09% reported in a large-scale cohort study from Shanghai [13]. The observed discrepancy stems from our study’s exclusive focus on newborns, unlike the Shanghai cohort which encompassed a broader age range (0–97 years). In that study, the authors documented an average annual hearing threshold decline of 0.4 dB/year with age, which directly accounts for the higher failure rate in their population. No significant difference in screening failure rates was observed between the homozygous and compound heterozygous mutation groups (35.54% vs. 31.19%, *p* = 0.357), aligning with the findings by Yu et al. that found no statistically significant difference in screening failure rates between the c.109G>A homozygous and c.235delC/c.109G>A compound heterozygous mutation groups (78.79% vs. 70%) [14]. Furthermorbilateral screening failure was the predominant pattern among screening failures, accounting for 82% of cases. This finding aligns with reports from Beijing and Tianjin [15,16], reinforcing that bilateral involvement is a characteristic feature of the Chinese population.

Notably, among the 425 newborns who passed the initial hearing screening, 1.88% (8/425) were subsequently diagnosed with hearing loss at 2.5–6 months of age, suggesting the possibility of either delayed-onset hearing loss or a false-negative initial screening result. This risk of missed or delayed diagnosis underscores the potential of genetic screening to improve detection, as evidenced by a meta-analysis demonstrating that combining genetic with conventional screening could identify an additional 13% of children with hearing impairment [17]. Strikingly, the benefit observed in our cohort is substantially greater, with genetic screening identifying an additional 21.62% (8/37) of cases, representing a figure nearly 1.7 times that reported in the meta-analysis. Multiple studies have established that GJB2 mutations confer a risk for delayed-onset or progressive hearing loss [6,13,18]. This pattern is exemplified by the work of Wu et al. [18], who reported that 22.64% of children with the c.109G>A homozygous mutation developed hearing loss upon follow-up to 2 years of age. To mitigate the risk of intervention delays due to false-negative results, it is advisable for newborns carrying GJB2 biallelic mutations to undergo diagnostic ABR testing within 3 months of age, irrespective of initial screening outcomes. Active monitoring at 12, 24, and 36 months is further suggested to ensure timely identification of any hearing deterioration.

In this study, the majority of confirmed hearing loss cases exhibited mild-to-moderate impairment (87.5%). Among the homozygous mutation group, 7 ears were diagnosed with profound deafness, all of which carried either the c.109G>A or c.235delC homozygous genotype. While the c.109G>A variant has been widely reported to be associated with mild-to-moderate phenotypes, its involvement in profound hearing loss as observed in our cohort and supported by other studies confirms that it can also lead to more severe outcomes, possibly due to the influence of genetic modifiers or environmental factors [19,20]. Functionally, the c.109G>A (p.V37I) variant leads to a partial loss of gap junction channel function, whereas c.235delC results in a complete loss. Theoretically, the c.109G>A/c.235delC compound heterozygous genotype (“partial + complete loss”) would be expected to produce more severe hearing impairment than the c.109G>A homozygous genotype (“partial + partial loss”). However, our study found no significant differences in hearing loss incidence (7.00% vs. 6.42%, *p* = 0.884) or severity between these two groups. This observation is consistent with findings reported by Ruan et al. (2024) in a Beijing neonatal cohort [13], though the limited sample size in both studies warrants further validation in larger populations.

In this study, hearing loss was classified into four grades according to the 2021 World Health Organization (WHO) hearing loss grading standard, based on auditory brainstem response (ABR) thresholds: mild (20–35 dB nHL), moderate (35–65 dB nHL), severe (65–80 dB nHL), and profound (80–95 dB nHL). To improve comparability with prior research, we systematically compared this framework with the criteria adopted in earlier GJB2-related studies. For instance, Snoeckx et al. [19]. defined hearing loss based on behavioral pure-tone average thresholds as mild (21–40 dB HL), moderate (41–70 dB HL), severe (71–95 dB HL), and profound (>95 dB HL). In line with Chen et al. [13] and other East Asian GJB2 studies, we employed ABR as the primary assessment method for infant hearing, given its objectivity and reliability in populations unable to cooperate with behavioral testing. It is important to note that threshold values differ across studies. For example, the upper limit for “severe” hearing loss in our study (80 dB nHL) is lower than that in the Snoeckx criteria (95 dB HL). This discrepancy arises largely from fundamental differences in the physiological basis and measurement dimensions between ABR and behavioral audiometry. Such comparisons highlight the importance of applying methodology-appropriate and internationally harmonized standards in pediatric hearing research, especially for infant populations. Adopting WHO standards promotes the integration and cross-comparison of data across different GJB2 cohorts, thereby establishing a more robust basis for accurately elucidating genotype–phenotype correlations.

In summary, this study systematically characterizes the audiological phenotypes and mutational spectrum of GJB2 biallelic mutations in newborns, elucidating the clinical heterogeneity of the c.109G>A and c.235delC variants. By demonstrating the value of combined genetic and dynamic audiological assessment, it provides an evidence-based rationale for refining early management strategies. Further validation with expanded sample sizes and prolonged follow-up is imperative to fully elucidate genotype-phenotype relationships and hearing loss progression.

## Figures and Tables

**Table 1:** Genotype distribution in 652 newborns with biallelic GJB2 mutations.

Genotype	Biallelic Mutations	Number of Cases (n)
Homozygote	c.109G>A/c.109G>A	537
	c.235delC/c.235delC	6
Compound Heterozygote	c.109G>A/c.235delC	83
	c.109G>A/c.176_191del16	3
	c.109G>A/c.176_191del16/c.538C>T	1
	c.109G>A/c.299_300delAT	15
	c.109G>A/c.427C>T	1
	c.109G>A/c.512insAACG	2
	c.235delC/c.176_191del16	1
	c.235delC/c.299_300delAT	2
	c.235delC/c.35delG	1

**Table 2:** Hearing screening results of the infants with biallelic GJB2 mutations.

Group	Number of Cases (n)	Hearing Screening Pass [n (%)]	Hearing Screening Failure [n (%)]	
			Unilateral Failure	Bilateral Failure
Homozygote	543	350 (64.46%)	33 (6.08%)	160 (29.46%)
Compound Heterozygote	109	75 (68.81%)	8 (7.34%)	26 (23.85%)

**Table 3:** Hearing Diagnosis Results of the infants with biallelic GJB2 mutations.

Group	Number of Cases (n)	Normal Hearing [n (%)]	Hearing Loss [n (%)]	
			Unilateral	Bilateral
Homozygous mutation	543	505 (93.00%)	8 (1.47%)	30 (5.53%)
Compound heterozygous mutation	109	102 (93.58%)	2 (1.83%)	5 (4.59%)

**Table 4:** The Results of Hearing Screening and Diagnosis.

Group	Number of Cases	Newborn Hearing Screening (n)	Hearing Diagnosis [n (%)]	
			Hearing Loss	Normal Hearing
Homozygous mutation	543	Passed: 350	6 (1.71%)	344 (98.29%)
		Failed: 193	32 (16.58%)	161 (83.42%)
Compound heterozygous mutation	109	Passed: 75	2 (2.67%)	73 (97.33%)
		Failed: 34	5 (14.71%)	29 (85.29%)

**Table 5:** The Results of the Severity of Hearing Loss.

Group	Number of Ears with Hearing Loss	Mild	Moderate	Severe	Profound
Homozygous Mutations	68	47	12	2	7
Compound Heterozygous Mutations	12	8	3	1	0

**Table 6:** Association of GJB2 Genotypes with Hearing Screening Results and Audiological Diagnostic Outcomes.

Genotype	Hearing Screening (n)		HL (n)	Ears with HL(n)			
	Passed	Failed		Mild	Moderate	Severe	Profound
c.109G>A/c.109G>A	348	189	35	45	12	1	4
c.235delC/c.235delC	2	4	3	2	0	1	3
c.109G>A/c.235delC	61	22	4	6	0	0	0
c.109G>A/c.176_191del16	3	0	0	0	0	0	0
c.109G>A/c.176_191del16/c.538C>T	1	0	0	0	0	0	0
c.109G>A/c.299_300delAT	8	7	0	0	0	0	0
c.109G>A/c.427C>T	0	1	1	2	0	0	0
c.109G>A/c.512insAACG	1	1	0	0	0	0	0
c.235delC/c.176_191del16	0	1	1	0	2	0	0
c.235delC/c.299_300delAT	0	2	1	0	1	1	0
c.235delC/c.35delG	1	0	0	0	0	0	0

## Data Availability

All data relevant to the study are included in the article.
